# Improving Cleaning Efficiency through the Measurement of Food Fouling Adhesive Strength

**DOI:** 10.1021/acsomega.4c00576

**Published:** 2024-05-13

**Authors:** Didem Sözeri Atik, Ibrahim Palabiyik, Goksel Tirpanci Sivri, Suzan Uzun, Yusuf Koç, Kübra Çalışır

**Affiliations:** † Department of Food Engineering, Tekirdağ Namık Kemal University, Tekirdağ 59030, Turkey; ‡ ARÇELİK A.Ş. R&D Center, İstanbul 34445, Turkey

## Abstract

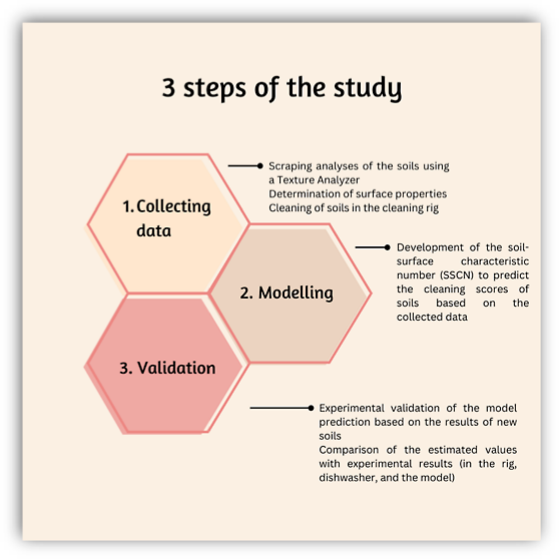

This study aims to investigate the impacts of factors, including textural properties, surface roughness, and contact angle, on the cleaning performance of food soils and develop a preliminary mathematical model to predict the cleaning score, depending on the soil-surface properties. The force required to remove soil from the surface was determined by a texture analyzer equipped with a newly designed probe. Potato puree and egg yolk soils showed high adhesive forces compared to other deposits. Margarine required the lowest force to detach from the surfaces. A soil-surface characteristic number (SSCN) was constructed from the results of contact angle, roughness, and textural analysis to predict the cleaning score depending on the soil-surface properties. The experimental work presented indicates that a higher SSCN was associated with lower cleaning scores for soil-surface combinations. Furthermore, a predictive model was developed to define the relationship between cleaning scores and SSCN. The applicability of the model was validated by measuring the cleaning performance of caramel and pudding soils on glass, porcelain, and stainless-steel household surfaces by using an automatic method. Therefore, it can be concluded that the SSCN approach can be improved in further studies to predict cleaning scores of soil-surface combinations in the experimental rig or automatic dishwasher.

## 1 Introduction

Cleaning food soils from surfaces is a general problem in the domestic kitchen and food industry. Food soils are required to be cleaned from dishware surfaces in every household. In contrast, cleaning is considered an essential part of food processing, which ensures food safety and quality by removing the fouled layers. However, the removal of food soils is challenging due to the complex composition of food and intense food processing applications such as high-temperature heat treatments for a long time.^1^ The success of cleaning depends on numerous factors, especially the properties of food soil, the interaction of soil with the surface, and the parameters of the cleaning process.^1−5^

Understanding the interactions between soil and surfaces and the connections involved in the soil is critical to perform successful cleaning. For instance, in food soil formation, food components either bind to the surface with adhesive forces or attach to other components on the surface with cohesive forces by forming a layer.^2,6,7^ The adhesion of the soil to the surface is associated with van der Waals, ionic, and electrostatic forces and is also dependent on the contact area, where the larger area results in greater attractive forces.^7,8^ The cohesive strength is related to the nature of the food soil, such as covalent bonds between food components.^7^ In cleaning, both cohesive and adhesive forces must be overcome to remove the soil from the surface. Therefore, many methods have been developed to quantify the forces involved in cleaning food soils.^9−14^

Zhang et al.^15^ developed the micromanipulation technique to measure the force required for disruption of the soil adhesion, which measures the bursting strength of cells. Then, this technique was modified to study both the adhesive and cohesive forces involved in food soils.^14^ In the micromanipulation device, a probe moves across the soiled surface, removing soil by scraping, and the adhesion force is calculated from the force required to detach the soil. Food soils such as starch,^7^ milk, whey protein,^16^ and tomato paste^14,17^ have been studied with micromanipulation to understand the interactions within the soil and between the soil and surface. The other technique used in the identification of the adhesion of soils is atomic force microscopy (AFM). AFM has been used to visualize the topography of surfaces and quantify the adhesion forces of cells,^18−20^ biofilms,^21^ and foodstuffs^1,9,22−24^ to surfaces.

The aforementioned empirical methods have been successfully applied to measure the forces required to detach the selected food soils. However, all of these approaches have only focused on measuring the forces required to remove the soil from the surface. It is crucial to link the measured forces to dishwasher cleaning, primarily applied in real life to clean food soils from surfaces. Texture profile analysis has been widely used to measure food properties, such as adhesiveness, cohesiveness, hardness, crispiness, and softness. The relationship among force, distance, and time provides valuable information about the textural properties of food. Texture analysis has the potential to develop a new model that enables the prediction of the optimum conditions for cleaning food soils from surfaces when it is linked to surface properties. For example, in cleaning, soil detachment from the surfaces is mainly related to overcoming the soil-to-soil (cohesive) and/or soil-to-surface (adhesive) interactions, which could be determined with texture analysis. Therefore, if texture analysis is applied to soils, the force response of soil can be used to establish a predictive model from the factors affecting the cleaning process for soils, particularly in dishwashers. Dishwasher cleaning programs apply preset temperatures, amounts of water, detergent, and cleaning durations for all soils, surface materials, and soil loads without knowing the difficulty of cleaning the soil; this leads to the loss of energy, water, and time. The development of a predictive model from the correlation of surface properties with texture analysis data presenting the force required to remove soil from the surface may contribute to the design of sustainable, smart, and automatic dishwashers that work efficiently and save considerable amounts of water, energy, or time.

The present work aims to develop a predictive model from surface properties (roughness and contact angle) and mechanical properties of soiled surfaces (force required for soil removal from the surface) and validate the applicability of the model to the dishwasher cleaning process. Furthermore, surface roughness and contact angle analyses were conducted for stainless steel, plastic, porcelain, and glass surfaces to understand the effect of surface properties. All surfaces were soiled with six food soils, which were different in physicochemical properties and representative of the most common food soils in dishwasher cleaning. The strength of interactions between the soil and surface was determined with a customized probe for texture analysis. The findings were attempted to be used to establish a predictive model for cleaning soil in situ and in a dishwasher system. Finally, the applicability of the model was validated with dishwasher cleaning experiments.

## 2 Materials and Methods

### 2.1 Materials

Stainless steel (SS 303 stainless steel no. 4 finish), glass, and porcelain surfaces were used, since they are the primary food contact materials that need to be cleaned. Surface samples were cut into 5 × 5 cm squares, which perfectly fit the holder for texture analysis and the cleaning process in the cleaning rig, as explained in the previous study by Palabiyik et al.^25^ As advised in the study of Heidrich et al.,^26^ commercial products, including spinach, egg yolk (EY), milk, potato puree (PP), minced meat, and margarine, which reflect consumer use, were purchased from a local market except for spinach and minced meat provided by Arçelik company. Standard porcelain dinnerware, stainless steel cookware sets, and glasses were used for the cleaning process in a dishwasher (Beko OlricDNM, Istanbul, Turkey). The standardized phosphate-free detergent of IEC 60436 and tap water were used to prepare the cleaning solution in both the cleaning rig and the dishwasher.

### 2.2 Methods

#### 2.2.1 Preparation of Soiled Surfaces

The international standard (59A/202/FDIS), which defines the amount of soil, the oven temperature, and the waiting time in the oven, was used for the soil preparation procedure determined by the International Electrotechnical Commission.^27^ In order to determine the adhesion forces between soil and surfaces, three different surfaces (metal, glass, and porcelain) were soiled with six different food products (minced meat, milk, spinach, EY, PP, and margarine). Also, new soils, namely, caramel and pudding, on three types of surfaces (glass, porcelain, and stainless steel) were prepared for the experimental validation of the model prediction used in the study. For this purpose, a commercial pudding product was purchased from a local market and caramel was traditionally produced by heating sugar. The soil was prepared on the surface as it would be 10 × 10 mm square for texture analysis and dried at 80 °C for 2 h. Also, the weight of soils used in the study is very low and does not exceed 0.05 Force (kg). Therefore, gravitational effects remain insignificant for scraping analyses.

Furthermore, for the cleaning process in the cleaning rig, soils were prepared according to the previous study.^25^ The soils were placed on surfaces through the flow line of the cleaning solution. Then, they were left to dry in the incubator at 80 °C for 2 h, and the cleaning process was conducted according to the study of Palabiyik et al.^25^

**Figure 1:**
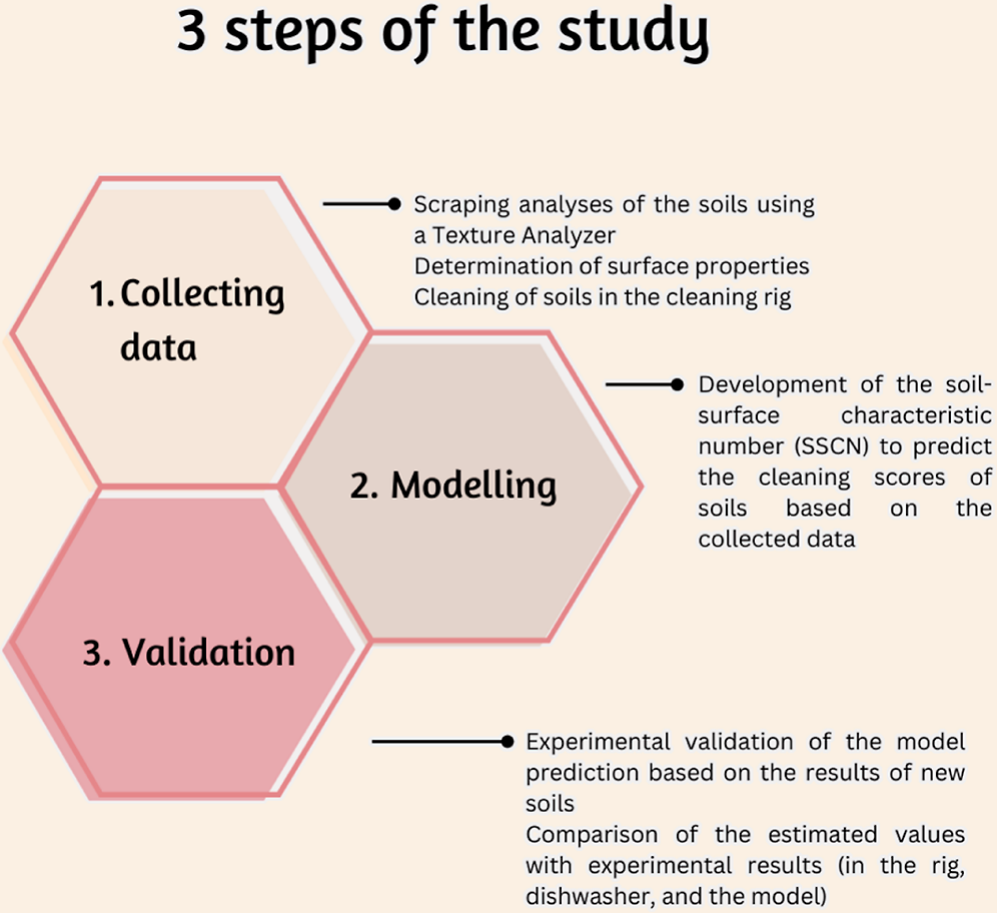
Outline of the study.

For the dishwasher cleaning process, soils were prepared according to the same standard, but soiling was applied to the dinnerware set, including plates, bowl mugs, and glasses, and the cookware set, including pots, saucepans, and frying pans, as seen in Figure S1. The evaluation chart of the cleaning performance is given in Table S1. The results are given as the mean and standard deviation of replicates. Also, three steps of the study are given in Figure 1 to clarify the process of the study.

#### 2.2.2 Probe Design for Texture Analysis

**Figure 2:**
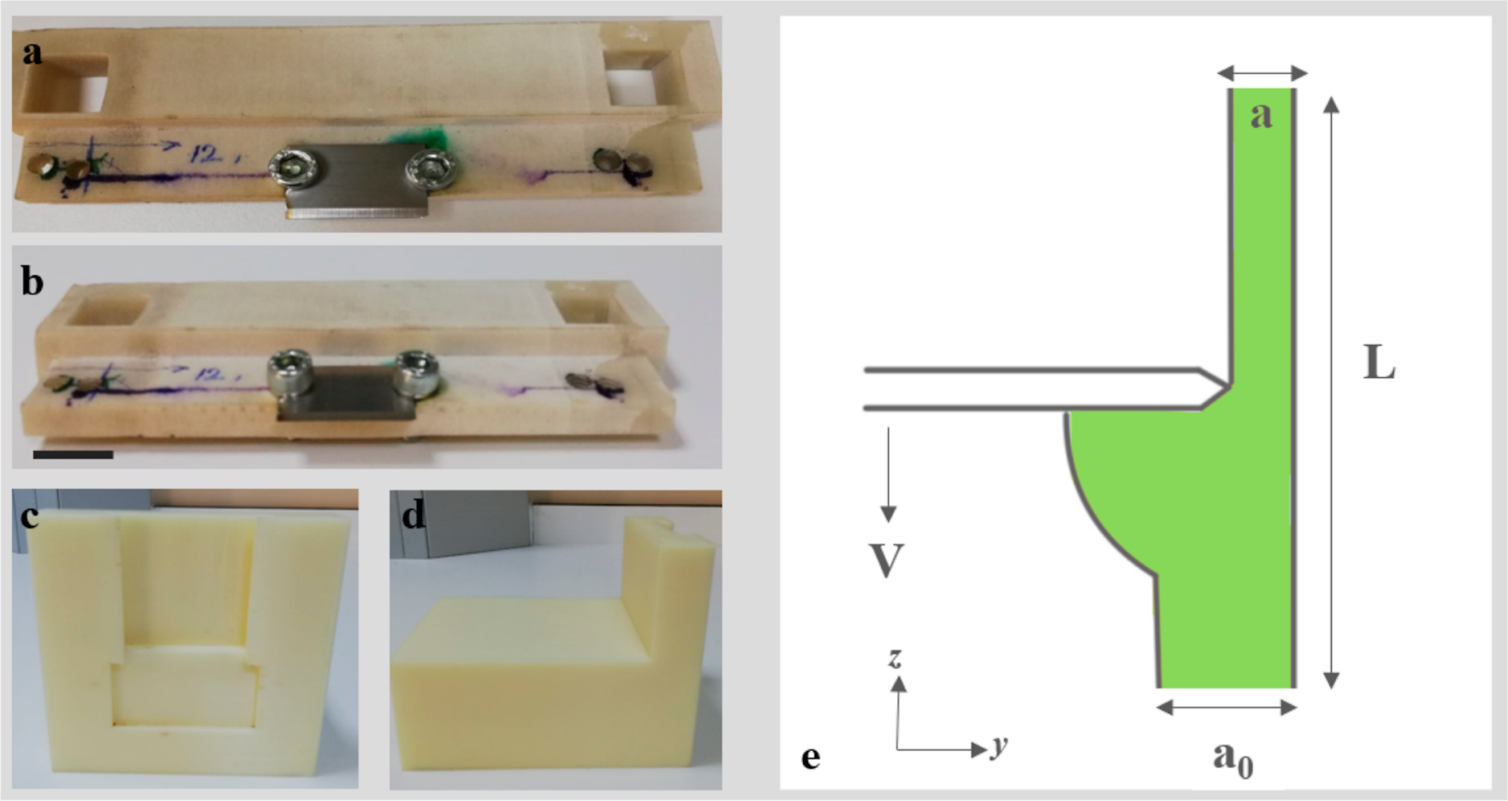
Schematic representation of the blade and holder (a,b): the top and side views of the scraping blade; (c,d): the holder for the soiled surfaces is placed for the texture analysis; and (e): the sketch of the scraping process (V: the constant speed of the blade, a: the thickness of soil after scraping, a_0_: the initial thickness of soil, and L: the length of soiled layer on the surface).

**Figure 3:**
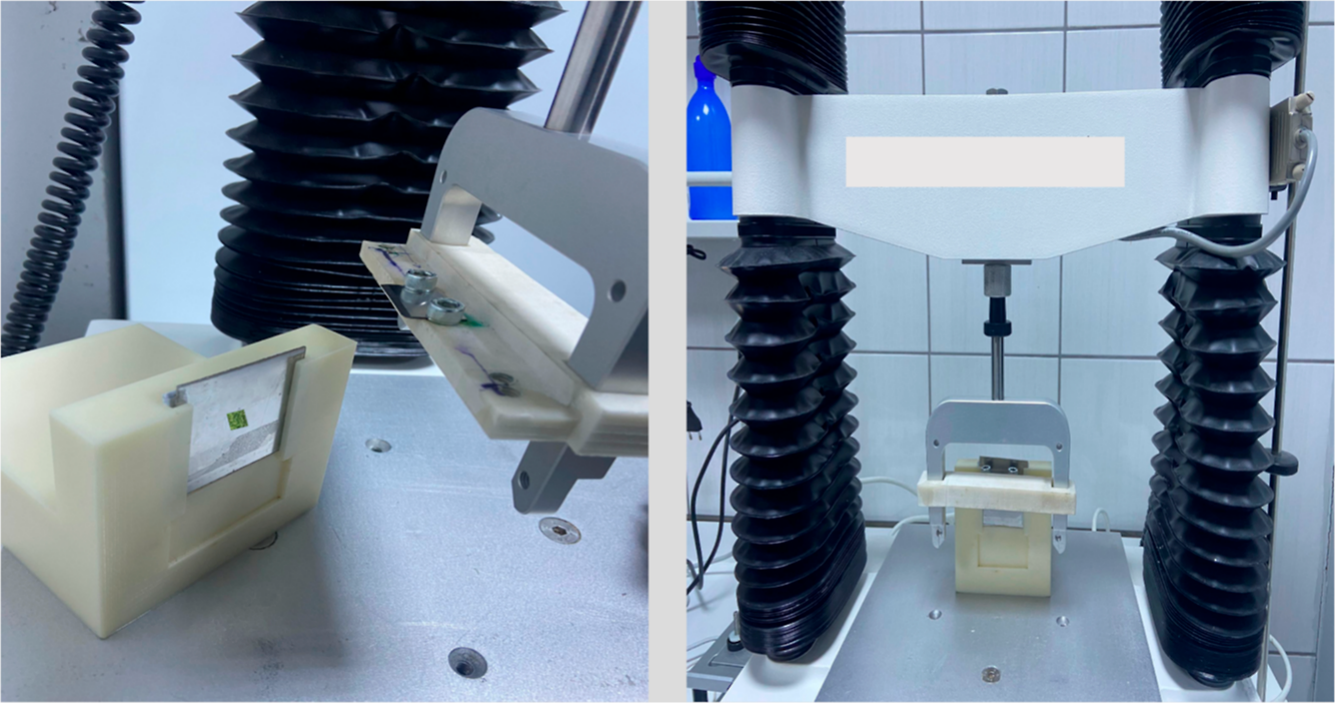
Schematic representation of texture analysis mechanism.

An experimental texture analyzer mechanism was designed to determine the force required for removing soils from surfaces. The designed blade and the sketch of the scraping process are shown in Figure 2. As seen in Figure 3, the designed scrapper was attached to the “extended craft knife” probe of the TA HD plus texture analyzer. The soiled surfaces were placed parallel to the scrapping blade with the help of a holder to simulate the scraping of the soils. The lengths of the blade and the soiled layer were 2 cm and 10 mm, respectively. The offset between the blade and surface was set to 0.1 mm to prevent frictional effects.

#### 2.2.3 Scraping Process of Soils

The soiled surfaces were placed in the holder. Then, the texture analyzer was set to a “return to start” cycle, a test speed of 2.0 mm/s, a post-test speed of 10 mm/s, and a distance of 10.0 mm. A force/time plot was made for every soiled surface, and the average force was calculated as the required force to exceed the adhesive forces between the soil and surface. Analyses were performed in triplicate, and the results are expressed as the mean and standard deviation values.

#### 2.2.4 Surface Roughness Measurement

Roughness measurements were performed on each surface using a profilometer (Veeco; Dektak 8) with a cutoff value of 1 mm to investigate the properties of surface samples. In order to accurately characterize the surface roughness, each sample was measured three times at different locations evenly distributed on each surface, and mean *R*_a_ values were obtained for each sample. The surface roughness values were recorded in graphical forms, shown in Figure S2.

#### 2.2.5 Measurement of Contact Angle

The contact angle of the surface samples was measured by an Attension Theta optical tensiometer (Biolin Scientific, Sweden/Finland). Attention Theta is used for the investigation of the material properties. The method was performed by applying single water droplets to the chosen samples and monitoring the contact angle. Ultrapure water with a volume of 10 μL was used as the experimental working medium at ambient temperature. The contact angle was measured between the sample surface and the liquid based on Young’s Laplace equation. The droplet was monitored by a video camera during measurements to make sure that the droplet was stabilized.^28^

#### 2.2.6 Cleaning Test Procedure

After the soiling process of the 5 × 5 cm square surfaces, they were placed into the cleaning rig, and the cleaning process was applied, as mentioned in the study by Palabiyik et al.^25^ The cleaning solution temperature was kept at 70 °C during the process. The cleaning time was restricted to 80 min, the maximum cleaning time for household dishwashers. For the cleaning process in the dishwasher, soiled utensils, including plates, mugs, and pots, were loaded into a dishwasher as stated in the standard of electric dishwashers for household use.^27^ The placement of the soiled utensils in the dishwasher was indicated in Figure S3. The economic program set up at 50 °C was chosen because it is the most preferred program in the dishwasher for household use.

#### 2.2.7 Model Development

A regression analysis was performed to understand the relationship between the properties of soiled surfaces and cleaning performance. Predicting the cleanliness of the soiled surfaces after a standard cleaning process is essential for dishwasher designers. To make regression analysis, a model described as a function with a “shoulder” period was used because it was observed that soiled surfaces having soil-surface characteristic number (SSCN) up to a critical level were cleaned with a maximum score (5). A soil-surface number was calculated from surface properties (roughness and contact angle) and mechanical properties of soiled surfaces (force required for soil removal from the surface) for each type of soil-surface pair. Sigma Plot 14.0 (Systat Software Inc., Chicago, IL, USA) was used for nonlinear regression analysis and to determine the parameters of nonlinear models. The goodness of the fit of the models was assessed using the regression coefficient (*R*_sqr_), adjusted regression coefficient (Adj. *R*_sqr_), and root-mean-square error (RMSE). The Adj. *R*_sqr_ measures how well a nonlinear model fits the data, and the higher the value, the better the adequacy of the model to describe the data. RMSE measures the average deviation between the experimental and fitted values. A small RMSE value of a model indicates a better fit of the data for that model.

## 3 Results and Discussion

### 3.1 Development of the Texture Analyzer Method for Measuring the Force Required to Disrupt and Remove Fouling Deposits

In our previous study,^25^ we investigated the influence of cleaning parameters on the cleaning performance of food soil from surfaces using a newly developed cleaning rig that mimicked a standard cleaning process in dishwashers. The results highlighted the importance of soil-surface interactions in the cleaning performance of soils. Further studies were required to determine the strength of soil-surface interactions for various soil-surface combinations and correlate it with surface properties to develop a predictive model for cleaning soil in situ and in real-time (dishwasher). In this study, an experimental texture analyzer mechanism has been developed to measure the force required for removing soils from surfaces. Figure 2 shows the parts of the experimental rig, and the top and side views of the scraping blade are given in sections a and b, respectively. The sketch of the scraping process was adapted from Tsai et al.^29^ The soiled surfaces are placed on a holder, as seen in parts c and d. The whole design of the texture analyzer can be seen in Figure 3. The setup uses the “extended craft knife” probe of the texture analyzer, and the mechanical removal is carried out using the TA HD Plus texture analyzer. Furthermore, the time at which the scraping will take place is determined to be the same as the time of preparation of the prepared soil. As a result of the textural analysis, a typical force–time curve was obtained, and the average force was recorded as the force required to remove the soil completely. Also, in the literature, a micromanipulation technique has been developed to determine the detachment force of soils from surfaces. A force transducer equipped with a T-shaped probe was used to measure the force as a function of time.^14^ In another significant study, Hooper et al.^17^ compared two techniques, namely, micromanipulation and fluid dynamic gauging, in baked tomato puree deposits. They reported that the two techniques depicted complementary information and the same trends. Furthermore, the study of Akhtar et al.^9^ compared AFM and micromanipulation techniques to determine the force required to detach deposits from surfaces. According to their results, both methods were valid in determining the relationship between the soils and surfaces. Another point worth noting is that a simple model was used to analyze the micromanipulation data in the study of Liu et al.^7^ They reported that the model needs to be expanded to account for a broader range of realistic failure mechanisms and the effect of changes in the cleaning conditions. In the present study, a texture analyzer, standard equipment for the food industry, was used to measure the force necessary to remove soil from surfaces.

### 3.2 Texture, Cleaning, and Surface Topography

**Figure 4:**
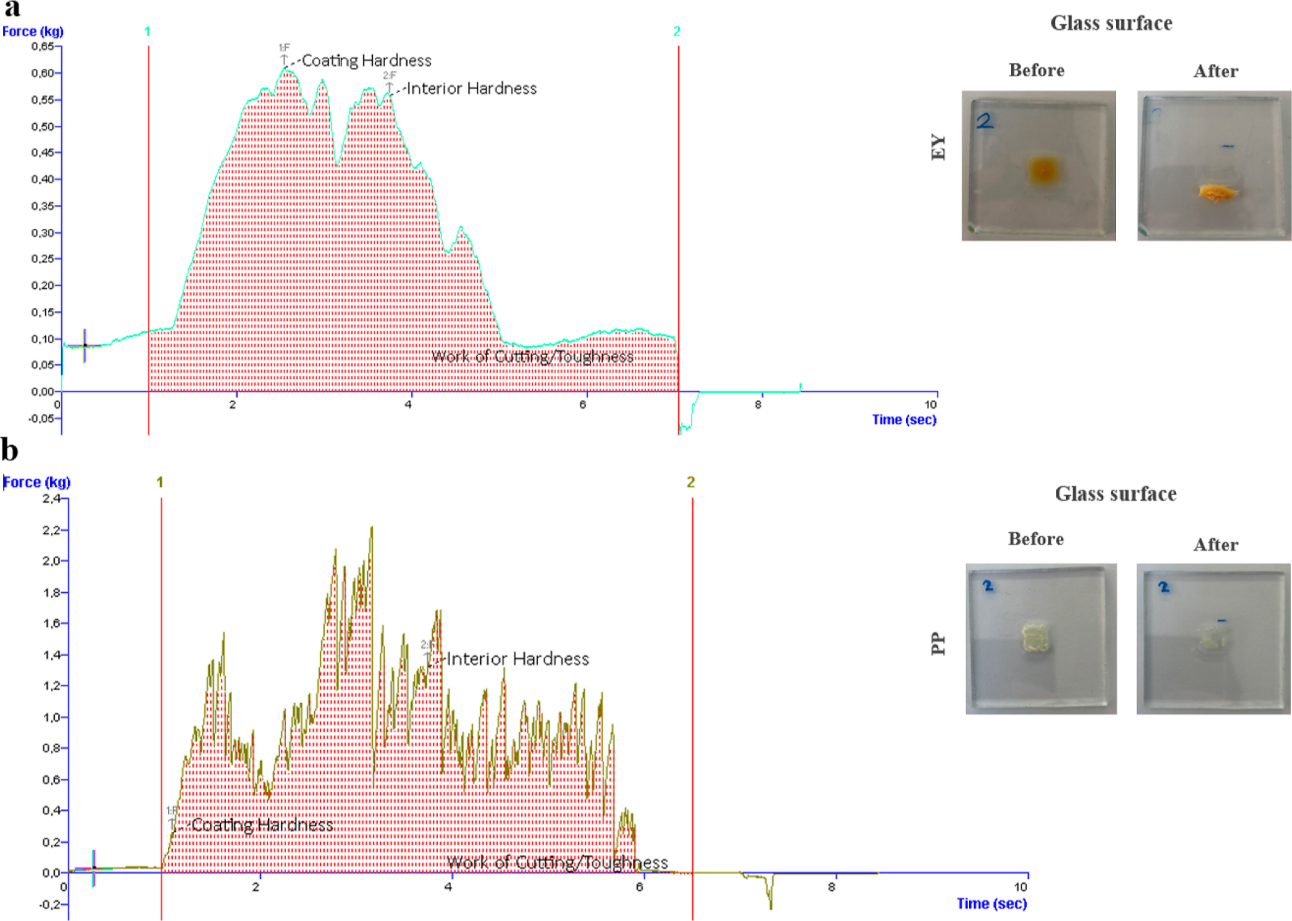
Force-time curves of EY (a) and PP (b) soils on the glass surface for the textural area measurements.

The results of texture analysis are shown in Figure 4 for EY and PP soils on the glass surface. It is apparent from Figure 4 that the profiles of the force–time curves are different based on the soil characteristics of the samples. Figure 4a shows that the homogeneous structure of EY soil resulted in a uniform graphic, whereas there are many peaks in Figure 4b for PP soil. It seems possible that these results are due to the particulate structure characteristics of PP soil when compared to EY soils, which have a smooth and gel-like structure. The images of different soil types can be seen before and after the texture analysis in Figures S4 and S5. According to the results, PP and EY soils showed high adhesive forces compared to those of other deposits, as shown in Table 1. On the other hand, the lowest force values required to detach soils were obtained from surfaces soiled with margarine. These results may be explained by the fact that the amount of fat in the soil decreased the power of interaction between the soil and surface, whereas high-starch content negatively influenced soil removal.^4^ Furthermore, a recent study observed that the fat content of the camel milk fouling deposit decreased with rising surface temperature.^30^ Moreover, the findings of the current study are consistent with those of Gordon et al.,^31^ who reported that EY soil was a complicated deposit to clean from surfaces. Therefore, it seems possible that these results stem from the denaturation temperature of the soils being lower than the drying conditions, which may cause the deposit to be more challenging to remove.^32^ The force required to detach milk soil from the surfaces was greater than those of spinach and minced meat soils. Changani et al.^33^ pointed out that various physicochemical factors were related to dairy-based fouling, such as pH change and calcium phosphate insolubilization, due to the reactions attributed to aggregation. The force required for the removal of minced meat soil was found to be between 324.00 and 3488.00 kg·m/s for different surfaces. A possible explanation for this might be that the fatty acid and protein-included soils are difficult to clean, as mentioned in the studies of Herrera-Márquez et al.^4^ and Snijders et al.^34^

**Table 1:** Results of Cleaning Six Different Soils on Three Surfaces at 70 °C, Texture Analysis, Roughness, and Contact Angle^a^

soil type	surface	experimental results of cleaning in the cleaning rig at 70 °C	average force (kg·m/s)	roughness (Ra, nm)	contact angle (degree)	SSCN
PP	G	5.00 ± 0.00	3556.00 ± 21.00	38.94	37.00	3742.44
PP	PO	5.00 ± 0.00	2626.00 ± 12.00	169.07	41.00	10828.73
PP	SS	4.50 ± 0.50	3433.00 ± 15.00	248.63	28.00	30483.81
M	G	5.00 ± 0.00	1069.00 ± 13.00	38.94	37.00	1125.05
M	PO	5.00 ± 0.00	2224.00 ± 80.00	169.07	41.00	9171.01
M	SS	5.00 ± 0.00	2362.00 ± 10.00	248.63	28.00	20654.04
EY	G	5.00 ± 0.00	3390.00 ± 26.00	38.94	37.00	3567.74
EY	PO	5.00 ± 0.00	3804.00 ± 12.00	169.07	41.00	15744.84
EY	SS	1.00 ± 0.00	4561.00 ± 12.00	248.63	28.00	40500.05
S	G	5.00 ± 0.00	913.00 ± 28.00	38.94	37.00	960.87
S	PO	5.00 ± 0.00	820.00 ± 11.00	169.07	41.00	3381.40
S	SS	2.50 ± 0.50	3987.00 ± 13.00	248.63	28.00	35403.13
MM	G	5.00 ± 0.00	324.00 ± 11.00	38.94	37.00	340.98
MM	PO	5.00 ± 0.00	375.00 ± 20.00	169.07	41.00	1546.37
MM	SS	4.00 ± 0.00	3488.00 ± 12.00	248.63	28.00	30973.72
MA	G	5.00 ± 0.00	394.00 ± 18.00	38.94	37.00	414.65
MA	PO	5.00 ± 0.00	230.00 ± 16.00	169.07	41.00	948.44
MA	SS	5.00 ± 0.00	132.00 ± 5.00	248.63	28.00	1172.11

a Soils; PP: potato puree, M: milk, EY: egg yolk, S: spinach, MM: minced meat, MA: margarine; surfaces: G: glass, PO: porcelain, SS: stainless steel; SSCN: soil surface characteristics number.

According to the study by Von Rybinski,^35^ several factors can influence the performance of cleaning, for instance, surface properties, hydrodynamic forces, soil type, temperature, time of cleaning, and detergent. One of the factors that determine the strength of the adhesion force is the surface properties, which are effective in adhesion of the soil. Furthermore, the contact area and the location of deposits influence the pull-off forces.^9^ The roughness and contact angle values of the surfaces are given in Table 1. It can be seen from the data that the contact angle values were reported to be 37, 41, and 28°, whereas roughness (RA) was found to be 38.94, 169.07, and 248.63 nm for glass, porcelain, and stainless-steel surfaces, respectively. Also, illustrations of the 3D roughness of the surfaces are shown in Figure S2. In addition, to observe the cleaning properties of soil and surface pairs, the experimental cleaning rig was used to obtain the cleaning results of six different soils on glass, porcelain, and stainless-steel surfaces.^25^ After cleaning at 70 °C for 80 min of soil surface combinations, the results of the present study indicate that high cleaning performances were achieved from margarine, milk, and PP soils for all surfaces. Also, the classification of soils based on their cleaning behavior is given in Table 2. The findings of the current study are consistent with those of Herrera-Márquez et al.,^4^ who reported high temperatures as the cause of melting fatty components in soil, making it effortless to detach from surfaces by the decline of adhesive forces. On the other hand, the EY was the most problematic soil to remove, especially on stainless-steel surfaces. Likewise, Pérez-Mohedano et al.^36^ and DuPont^37^ reported that EY soils, in particular, were one of the most difficult to detach. In addition, this material is challenging to remove from a hard surface when dried and is one of the most common complaints in the automated dishwasher industry.

**Table 2:** Classification of Soils Based on the Cleaning Behavior

adhesive failure	cohesive failure
milk	minced meat
margarine	spinach
egg yolk	potato puree

Moreover, in the present study, the cleaning performance of minced meat and spinach soils was five for all surfaces except stainless-steel plates, as small pieces adhered to the surface. These results can be attributed to the high roughness and low contact angle values of stainless-steel surfaces compared with glass and porcelain surfaces. So far, various grades and finishes of stainless-steel surfaces have been identified as potentially important factors in adhesion due to their different topographical properties.^38^ On the other hand, an exception emerged in only margarine soil in the present study. This result may be explained by the hydrophobic nature of fat-based soils. Similarly, Cuckston et al.^39^ reported that the existence of mobile fat could be a cause of a decrease in the adhesiveness of deposits. In addition, numerous studies reported that the lowest *R*_a_ values were in a positive relationship with surface cleanability.^40,41^ Therefore, in the present study, the observed difference in cleaning results between surfaces could be attributed to the significant role of surface topography in cleanability. However, it is not possible to explain or predict the cleaning behavior of soils based on limited parameters. For this purpose, it is essential to produce a preliminary model that includes all cleaning-related parameters to explain the cleaning properties of the soil surface pairs.

### 3.3 Development of the Soil-Surface Characteristic Number

Developing a model to predict cleaning behavior is advantageous for industrial applications to ensure cost-effectiveness. Numerous studies have attempted to explain the cleaning mechanism of several soils with experimental results by using gravimetric approaches,^42^ optical methods,^43^ micromanipulation measurements,^44^ and AFM.^9^ On the other hand, one of the limitations of these techniques is that one laboratory method does not give satisfactory results when characterized by the cleaning behavior of food soils. Predicting the cleaning scores of such a soil-surface pair requires knowledge of both soil and surface properties and a model for removing soil material. In the present study, data obtained from contact angle, roughness, and textural analyses were used to construct a SSCN. The results collected from the cleaning experiments showed that the cleaning efficiency was inversely proportional to the contact angle, which was directly proportional to the adhesion force and roughness. In eq 1, the equation of SSCN is presented, where the average force (force required to detach a soil) and roughness are in direct proportion, but the contact angle is in reciprocal proportion to the SSCN. According to the obtained results, the higher SSCN was associated with the lower cleaning scores of soil-surface combinations. The calculated SSCN values of all samples are given in Table 1.
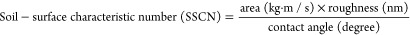
1

Furthermore, a predictive model was used to define the relationship between the cleaning results and SSCN, as given in eq 2. The model is described as a function with a “shoulder” period maximum cleaning score is observed up to a critical SSCN depending on the soil-surface properties to obtain the nonlinear regression between the soil-surface properties. Because the maximum limit of the cleaning score was five due to the cleaning evaluation procedure used in the study, the maximum cleaning score was fixed to five before performing the regression.
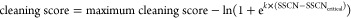
2in which the maximum cleaning score is the shoulder length [maximum cleaning score] and *k* is the rate constant.^45^

**Figure 5:**
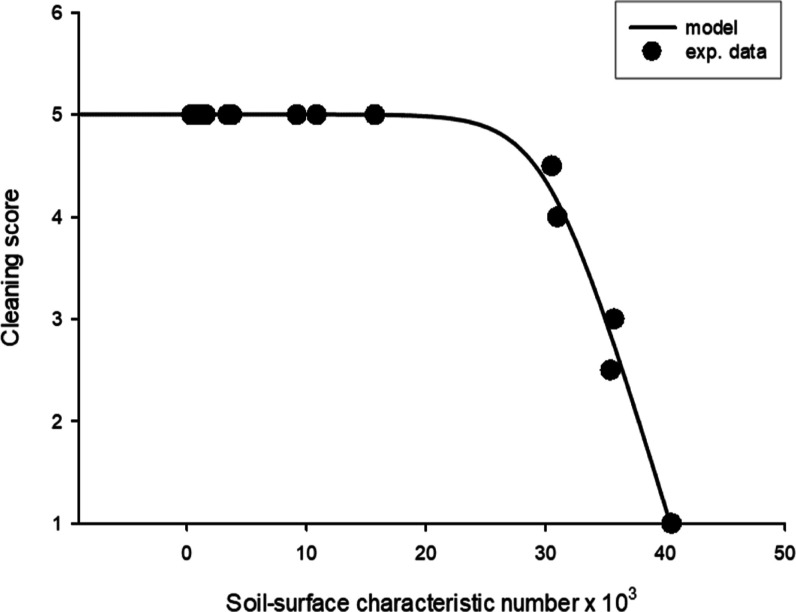
Data of the mathematical model and experimental results allow the quantitative description of cleaning kinetics.

Figure 5 presents the data of the mathematical model and experimental results, allowing for the quantitative description of cleaning kinetics. As shown in Figure 5, the cleaning scores do not progress until the critical SSCN (SSCN_critical_ = 30.212) but then begin to decrease with the increasing value of SSCN. The applied cleaning conditions in this study achieved the maximum cleaning performance until SSCN reached the critical level. However, after that, the adhesive forces between soil and surfaces surpassed. The goodness-of-fit of the models was evaluated using the *R* square and RMSE values, and the statistical results of the predictive model are given in Table 3. It is apparent from Table 3 that the model gave a good qualitative description of the experimental results. Recently, Helbig et al.^46^ used differential scanning calorimetry, rheology, optical swelling measurements, and micromanipulation analysis to reflect the complex interactions between soil and surface. According to the results, the micromanipulation method could predict the cleaning performance of EY soils. At the same time, diffusion, rheological analysis, and swelling were indicators of the cleaning behavior tendencies. Furthermore, as mentioned in the study of Herrera-Márquez et al.,^4^ the multiscale “cleaning map strategy” was constructed to display cleaning results, which permitted the selection of the most applicable conditions for cleaning. Subsequently, these current findings contribute to a growing body of literature on the cleaning score of the different soil-surface pairs, which can be predicted using measured surface and soil properties. However, in the present study, determining the average force (force required to detach a soil) value of soil-surface pairs has brought a different perspective to the texture analyzer with the ease of use and short-term prediction of cleaning performance by using the data of contact angle and roughness.

**Table 3:** Data of Mathematical Model to Predict SSCN

	coefficient	standard error	*t*	*P*
*y*_max_	5.0033	0.0373	134.0530	<0.0001
*k*	0.3909	0.0225	17.3921	<0.0001
*x*_critical_	30.2124	0.4220	71.5960	<0.0001
*R* square		0.9876		
adj. *R* square		0.9859		
RMSE		0.1348		

### 3.4 Experimental Validation of the Model Prediction

The predictive model was used to anticipate the cleaning performance of new soils, namely, caramel and pudding, on three types of surfaces (glass, porcelain, and stainless steel). The required input parameters to estimate the cleaning score of these soils are their average force for removing the soil from a surface as well as the roughness and contact angle of the surfaces to calculate SSCN. For this purpose, textural analyses of new soils (caramel and pudding) were conducted. According to the results, the highest adhesive forces were obtained at 6378.00 and 7794.00 kg·m/s on the stainless-steel surface for caramel and pudding, respectively; thus, these results were found to stay under the SSCN_critical_. A possible explanation for this could be that the stainless-steel surface has the highest roughness value and the lowest contact angle value, which resulted in a higher SSCN number. On the other hand, the lowest values of force required to remove soils were obtained from porcelain and glass surfaces soiled with pudding compared to caramel soils, as shown in Table 4. This finding agrees with the data of Akhtar et al.,^9^ which showed that different removal forces were obtained for identical soil from different surfaces. Also, according to their study, caramel soil had the greatest adhesion ability on stainless-steel surfaces compared with any other surface. Moreover, if temperatures are high enough to cause caramelization and polymerization of the sugars and fats, the confectionary deposits might exhibit strong adhesiveness to the test surface.^9^

**Table 4:** Experimental and Predicted Results of the Model^a^

soil type	surface	average force (kg·m/s)	SSCN	predicted cleaning score from the model	experimental results of cleaning in the cleaning rig at 70 °C	experimental results of cleaning in a dishwasher
P	G	4919.00	5177.57	5	5.00 ± 0.00	3.00 ± 0.00
P	PO	3694.00	15232.79	5	5.00 ± 0.00	4.00 ± 0.00
P	SS	7794.00	69207.936	1	1.50 ± 0.50	4.00 ± 0.00
CA	G	6128.00	6450.13	5	5.00 ± 0.00	3.00 ± 0.00
CA	PO	6054.00	24964.62	5	5.00 ± 0.00	4.00 ± 0.00
CA	SS	6378.00	56634.362	1	5.00 ± 0.00	4.00 ± 0.00

a Soils; P: pudding, CA: caramel; surfaces: G: glass, PO: porcelain, SS: stainless steel; and SSCN: soil surface characteristics number.

After the textural analyses, the SSCN for caramel and pudding soils was calculated using the force required to remove soils, contact angle, and roughness values of specimens. Subsequently, Figure 5 shows prediction of the cleaning performance of the soils. According to the prediction based on Figure 5, the highest cleaning score (5) should be achieved on the glass and porcelain surfaces for both soils after cleaning at 70 °C for 80 min. On the other hand, the estimated cleaning score from the model was one for both soils on the stainless-steel surface. The next step was to demonstrate the accuracy of SSCN in predicting the cleaning results of these soil surface combinations. For this purpose, the experimental cleaning rig was used to clean caramel and pudding soils from porcelain, glass, and stainless-steel surfaces at 70 °C. As can be seen from Table 4, the model provided a good qualitative description of the experimental results except for one soil surface combination, which is caramel deposit and stainless-steel surface. In this exception, the predicted cleaning score was determined as one, whereas the experimental cleaning result of the soil surface combination was found to be five. The findings from the current study regarding contact angle and roughness suggest that the stainless-steel surface may possess hydrophilic properties. As a result of this feature, the stainless-steel surface may hold sugar better in caramel soil. On the other hand, because there is water during the cleaning process, the cleaning score is higher because sugar dissolves easily in water.

After this stage, the cleaning performance of caramel and pudding soils on glass, porcelain, and stainless-steel household surfaces was measured by using an automatic dishwasher to demonstrate the applicability of SSCN and the model. As shown in Table 4, there are several differences between the predicted cleaning scores from the model and the experimental results of cleaning in an automatic dishwasher. According to the study by Wang et al.,^47^ the coverage provided by the water jets is thought to be an essential component in the cleaning effectiveness of a dishwasher. Also, the impinging jets in automatic dishwashers can impact the surfaces at various angles.^36^ On the other hand, in the present study, the cleaning solution flows directly over the soil in the cleaning rig. Therefore, it seems possible that these differentiations between scores might be due to the variety in the flow mechanism of the cleaning solution over surfaces. Also, the temperature was not stable during the cleaning process because of the standard dishwasher operating conditions, whereas the cleaning performance of the surfaces used in this study was observed at a fixed temperature. Furthermore, the usage frequency of surfaces can affect the applicability of the model since the surface properties of soiled materials can change over time. On the other hand, it is similar to the evaluation of dinnerware set by dishwasher producers. For future studies, perhaps a correction coefficient that takes into account the damage to the surfaces over time can be added to the model. Overall, the model and SSCN approach have proven to be a practical technique in this study, although it still requires more refinement to be more precise.

## 4 Conclusions

This study proposes a textural analysis technique to measure the required force to remove soils from different surfaces. Also, a SSCN was constructed from the contact angle, roughness, and textural analysis results. The predictive model, based on SSCN, was used to anticipate the cleaning performance of new soils, and the results of the estimated values, the cleaning in the experimental rig, and an automatic dishwasher were compared. One of the more significant findings from this study is a different area of use provided to a currently used laboratory device. The results of this research support the idea that a texture analyzer method is a convenient technique to measure the detachment force of soils from surfaces. The present study made several noteworthy contributions to cleaning studies, reporting that the SSCN approach can be an acceptable technique to predict cleaning scores of soil-surface combinations in the experimental rig or an automatic dishwasher to improve the efficiency of energy and water uses. Furthermore, further experimental investigations can be used to examine the possibility of adding surface energy to the equation for converting SSCN to a dimensionless number.

## Data Availability

The data that support the findings of this study are available on request from the corresponding author. The data are not publicly available due to privacy or ethical restrictions.

## Supporting Information Available

Sample pictures of soiled dinnerware set for the cleaning application in the dishwasher, evaluation of the cleaning performance (59*A*/202/FDIS), 3D roughness illustrations of the surfaces, placement of the soiled utensils in the dishwasher, and images of all soils before and after texture analysis (PDF)
