# Supplementary material for: Improving Cleaning Efficiency through the Measurement of Food Fouling Adhesive Strength
Source: ACS Omega. 2024 May 13;9(20):22156–65. doi: 10.1021/acsomega.4c00576 (PMC11112590; doi:10.1021/acsomega.4c00576)

Improving Cleaning Efficiency through the Measurement of Food Fouling Adhesive Strength

Didem Sözeri Atik[1*], Ibrahim Palabiyik[1], Goksel Tirpanci Sivri[1], Suzan Uzun[1], Yusuf Koç[2], Kübra Çalışır[2]

[1] *Department of Food Engineering, Tekirdağ Namık Kemal University, Tekirdağ, Turkey*

[2]*ARÇELİK A.Ş. R&D Center, İstanbul, Turkey*

dsozeri@nku.edu.tr, ipalabiyik@yahoo.com, gtirpanci@nku.edu.tr, suzanuzun@nku.edu.tr, yusufkoc3@gmail.com, clsrkubra@gmail.com

[*]Corresponding author: Didem Sözeri Atik, Department of Food Engineering, Tekirdağ Namık Kemal University, Tekirdağ, Turkey Tel. +905397820439

E-mail address: dsozeri@nku.edu.tr, didem.sozeri@yahoo.com

Figure S1. The sample pictures of soiled dinnerware set for the cleaning application in the dishwasher

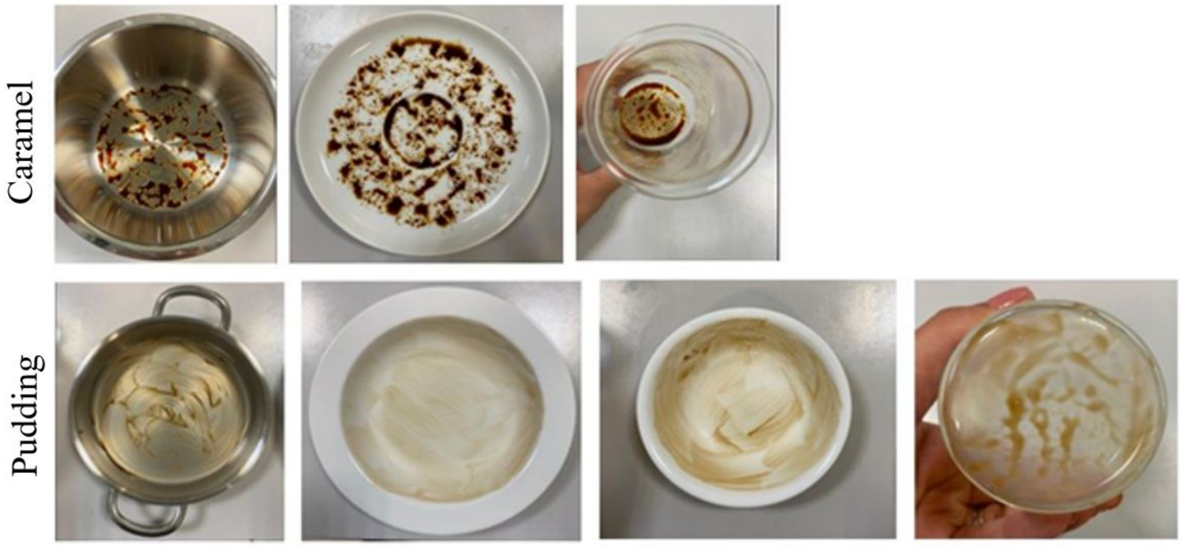

Table S1. Evaluation of the cleaning performance (59A/202/FDIS)*

<table>
<tr><th>Number of small dot shaped soil particles</th><th>Total solid area mm^2</th><th>Score</th></tr>
<tr><td>0</td><td>$A=0$</td><td>5</td></tr>
<tr><td>1-4</td><td>$0<A\leq 4$</td><td>4</td></tr>
<tr><td>5-10</td><td>$4<A\leq 20$</td><td>3</td></tr>
<tr><td>>10</td><td>$20<A\leq 50$</td><td>2</td></tr>
<tr><td>Not applicable</td><td>$50<A\leq 200$</td><td>1</td></tr>
<tr><td>Not applicable</td><td>$200<A$</td><td>0</td></tr>
<tr><td colspan="3">Each load item shall be awarded a score from the table according to the category of soil area or number of discrete soil particles adhering to the item. If the requirements for more than one score are met, the lowest applicable score shall be awarded.</td></tr>
</table>

*IEC 60436 4th Edition. (2015). Electric dishwashers for household use–methods for measuring the performance.

Figure S2. 3D roughness illustrations of the surfaces

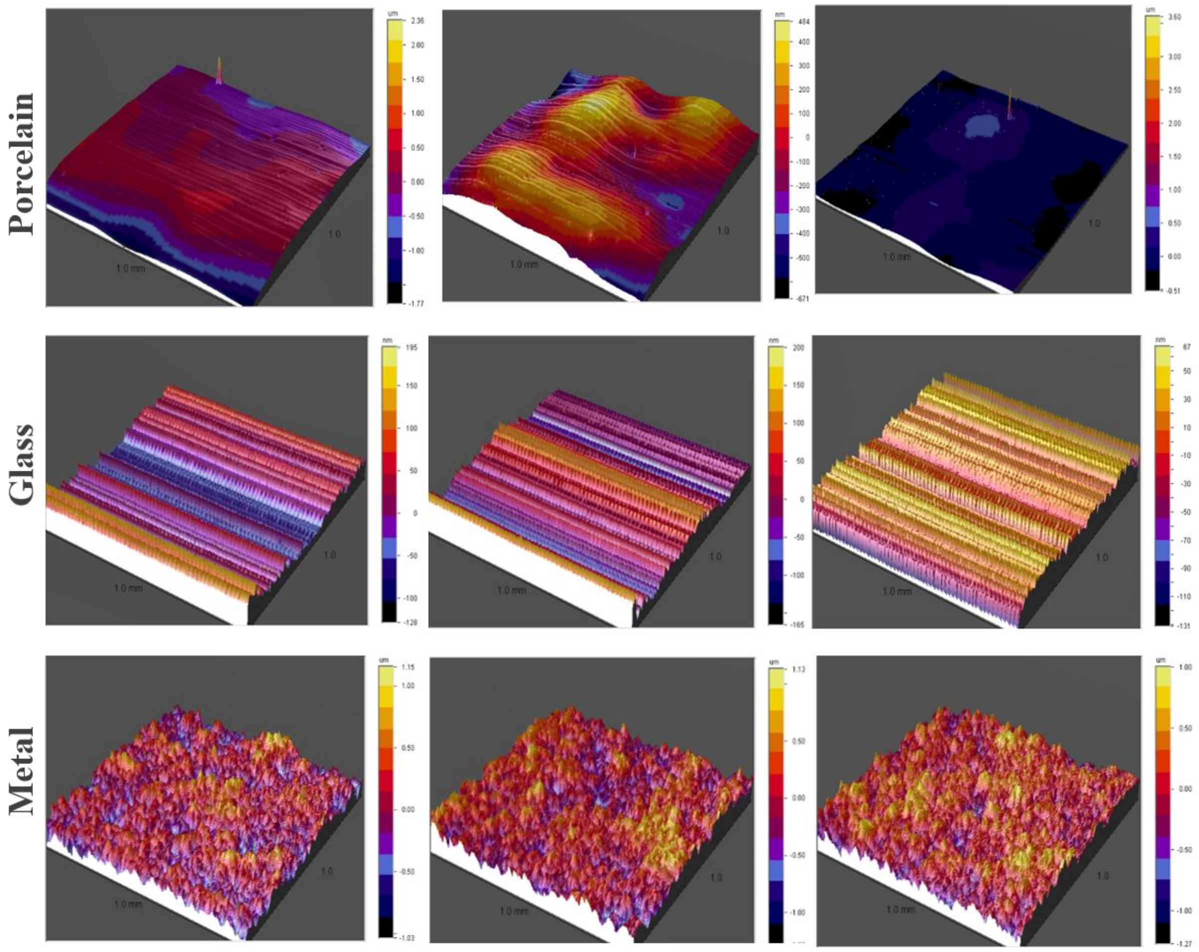

Figure S3. The placement of the soiled utensils in the dishwasher

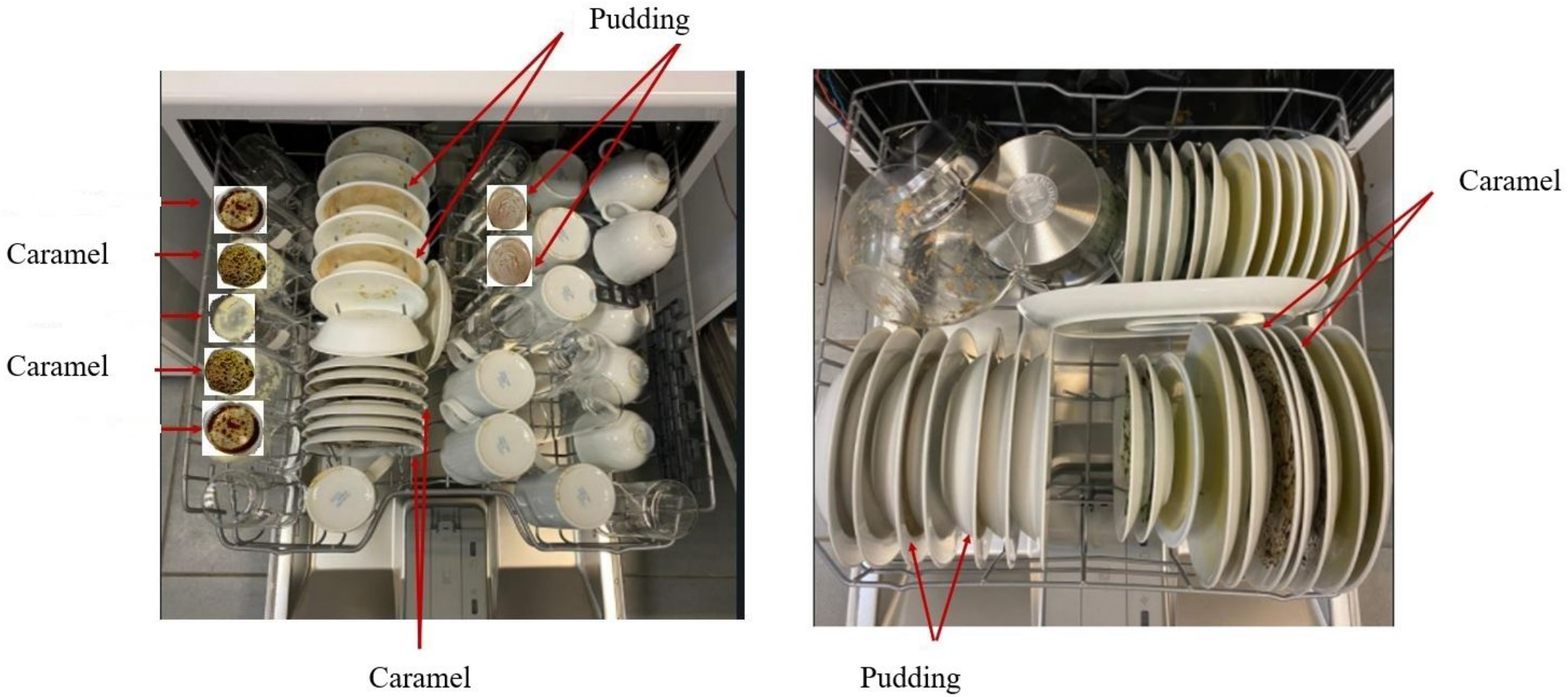

Figure S4.The images of PP, M, and EY soils before and after texture analysis (Soils; PP: potato puree, M: milk, EY: egg yolk; Surfaces: G: glass, PO: porcelain, SS: stainless steel)

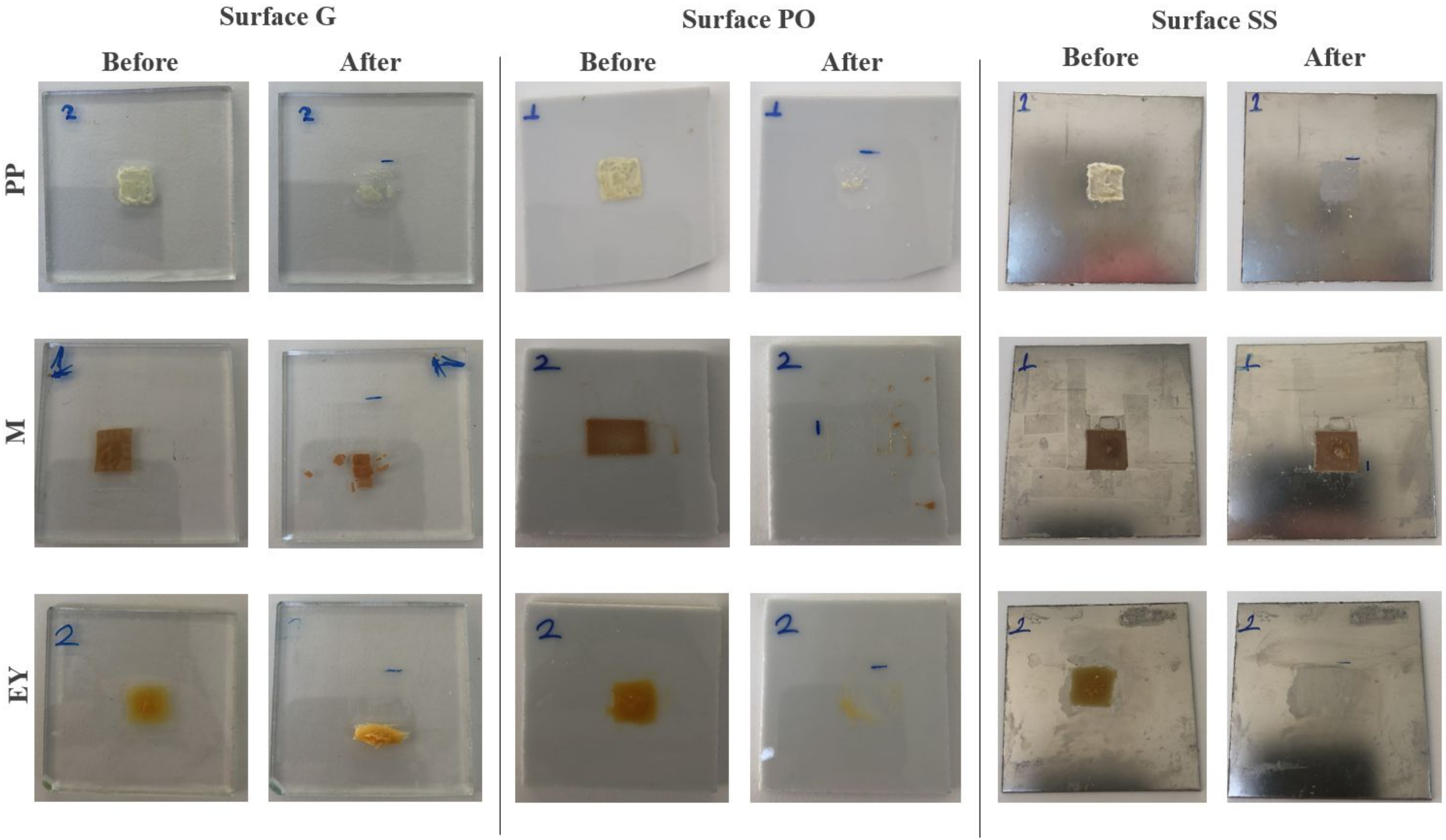

Figure S5. The images of S, MM, and MA soils before and after texture analysis *(Soils; S: spinach, MM: Minced meat, MA: margarine; Surfaces: G: glass, PO: porcelain, SS: stainless steel)

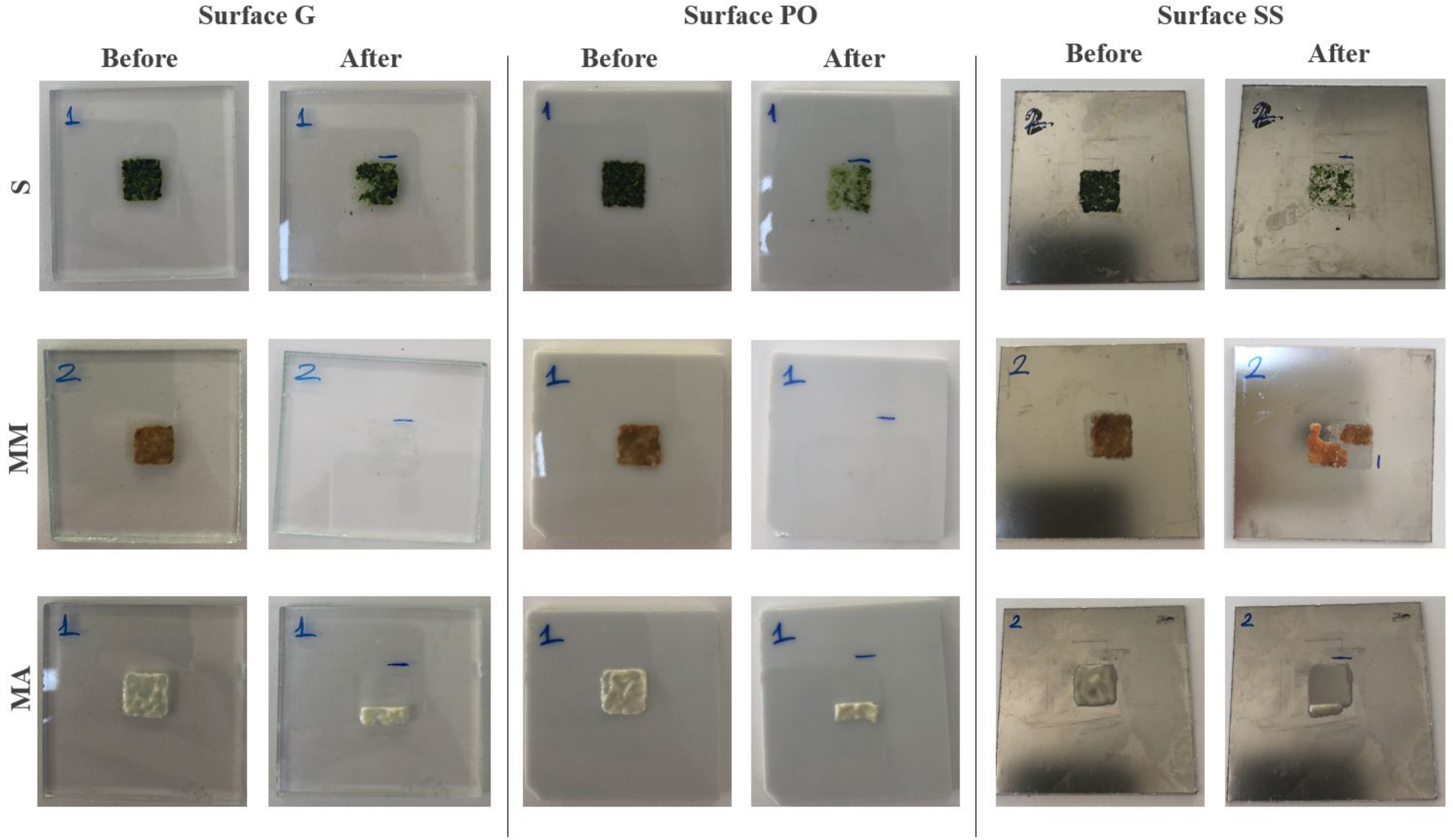

Supplement: Supplementary file 1 — ao4c00576_si_001.pdf [file ao4c00576_si_001.pdf]
